# Susceptibility and barriers to infection of Colorado mosquitoes with Rift Valley fever virus

**DOI:** 10.1371/journal.pntd.0009837

**Published:** 2021-10-25

**Authors:** Daniel A. Hartman, Nicholas A. Bergren, Therese Kondash, William Schlatmann, Colleen T. Webb, Rebekah C. Kading

**Affiliations:** 1 Department of Microbiology, Immunology, and Pathology, Colorado State University, Fort Collins, Colorado, United States of America; 2 Department of Environmental and Radiological Health Sciences, Colorado State University, Fort Collins, Colorado, United States of America; 3 Vector Disease Control International, Loveland, Colorado, United States of America; 4 Department of Biology, Colorado State University, Fort Collins, Colorado, United States of America; Oregon State University College of Veterinary Medicine, UNITED STATES

## Abstract

Rift Valley fever virus (RVFV) causes morbidity and mortality in humans and domestic ungulates in sub-Saharan Africa, Egypt, and the Arabian Peninsula. Mosquito vectors transmit RVFV between vertebrates by bite, and also vertically to produce infectious progeny. Arrival of RVFV into the United States by infected mosquitoes or humans could result in significant impacts on food security, human health, and wildlife health. Elucidation of the vectors involved in the post-introduction RVFV ecology is paramount to rapid implementation of vector control. We performed vector competence experiments in which field-collected mosquitoes were orally exposed to an epidemic strain of RVFV via infectious blood meals. We targeted floodwater *Aedes* species known to feed on cattle, and/or deer species (*Aedes melanimon* Dyar, *Aedes increpitus* Dyar, *Aedes vexans* [Meigen]). Two permanent-water-breeding species were targeted as well: *Culiseta inornata* (Williston) of unknown competence considering United States populations, and *Culex tarsalis* Coquillett as a control species for which transmission efficiency is known. We tested the potential for midgut infection, midgut escape (dissemination), ovarian infection (vertical transmission), and transmission by bite (infectious saliva). Tissues were assayed by plaque assay and RT-qPCR, to quantify infectious virus and confirm virus identity. Tissue infection data were analyzed using a within-host model under a Bayesian framework to determine the probabilities of infection outcomes (midgut-limited infection, disseminated infection, etc.) while estimating barriers to infection between tissues. Permanent-water-breeding mosquitoes (*Cx*. *tarsalis and Cs*. *inornata*) exhibited more efficient horizontal transmission, as well as potential for vertical transmission, which is contrary to the current assumptions of RVFV ecology. Barrier estimates trended higher for *Aedes spp*., suggesting systemic factors in the differences between these species and *Cx*. *tarsalis* and *Cs*. *inornata*. These data indicate higher potential for vertical transmission than previously appreciated, and support the consensus of RVFV transmission including a broad range of potential vectors.

## Author summary

Rift Valley fever virus (RVFV) causes morbidity and mortality in humans and domestic ungulates in sub-Saharan Africa, Egypt, and the Arabian Peninsula. Mosquito vectors transmit RVFV between vertebrates by bite, and also vertically to produce infectious progeny. To inform vector control priorities upon the introduction of RVFV to the United States, we tested the ability of three floodwater *Aedes* species known to feed on cattle and/or deer in Colorado (*Aedes vexans* [Meigen], *Aedes melanimon* Dyar, *Aedes increpitus* Dyar) to transmit RVFV (vector competence). We also tested *Culiseta inornata* (Williston), and *Culex tarsalis* Coquillett which exhibits high vector competence, and the potential for vertical transmission by testing ovaries. These data were modeled to estimate the potential for virus transmission, based on the infection probabilities of different organs that serve as transmission barriers inside the mosquitoes. The permanent-water-breeders *Cs*. *inornata* and *Cx*. *tarsalis* exhibited higher efficiency of horizontal transmission as well as potential vertical transmission. *Aedes* species were less efficient at vertical and horizontal transmission, with high barriers to infection of and dissemination from the midgut. Overall, these data support the transmission of RVFV by a broad range of potential vectors in the United States, posing a major challenge for vector control if this virus is introduced.

## Introduction

Rift Valley fever virus (RVFV) is a mosquito-borne virus (Order: *Bunyavirales*, Family: *Phenuiviridae*, Genus: *Phlebovirus*) endemic to sub-Saharan Africa that affects both humans and domestic ungulates [1]. Clinical signs in animals include spontaneous abortion, and near total mortality of neonatal ungulates, while human illness manifests as acute febrile illness, with low rates of encephalitis, hemorrhagic fever, and blindness [1,2].

While the epidemiology of RVFV is nuanced across its range, the importance of mosquito-borne transmission seems to be universal. While direct transmission of RVFV occurs between infected animals and humans, vector-borne transmission is critical to epizootics as well as interepidemic transmission [2]. In addition to horizontal transmission by mosquitoes, there is strong evidence for vertical transmission by some floodwater *Aedes spp*. mosquitoes [3,4]. These species oviposit dessication-resistant eggs which may be able to harbor virus throughout periods with little to no rainfall. This persistence in the mosquito population is thought to be a mechanism of viral maintenance, allowing the virus to survive long interepidemic periods in mosquito egg populations, which can hatch following periods of high rainfall [3,5]. Preliminary evidence for vertical transmission has been demonstrated under laboratory conditions using a colonized line of *Culex tarsalis* Coquillett [6]; however, the implications for vertical transmission by a permanent-water ovipositing mosquito are unclear for the epidemiology of RVFV. Vertical transmission of viruses in the vector is well-documented throughout the order *Bunyavirales* [7].

The first described RVFV epizootic event occurred on a sheep ranch near Lake Naivasha, Kenya, where abortion storms were observed among ewes, along with high mortality in lambs [8]. Subsequent epizootics and epidemics have been observed throughout the African continent, with notable expansions into Egypt [9], Madagascar [10], and Saudi Arabia [11,12], making RVFV an increasing emerging disease risk for other continents such as Europe and North America. RVFV is listed as an overlap select agent pathogen in the United States [13], and as such represents a biosecurity and bioterrorism threat.

The main potential introduction pathway to the United States is suspected to be human travel via airline [14]. Establishment of RVFV, however, requires the presence of competent vectors and amplification hosts, and the United States has both for RVFV [15]. White-tailed deer exhibit high RVFV titers upon infection [16], and some theoretical evidence exists regarding the competency of animals in the orders Artiodactyla, Lagomorpha, and Carnivora to serve as amplification hosts [15]. Overall, however, the data on vertebrate competence are lacking.

A wealth of work has been produced on vector competence of United States mosquitoes in the laboratory [17–20], although important gaps still exist for mosquitoes that feed on potential amplifying hosts for RVFV in the United States, such as white-tailed deer. This ecological context is imperative to assessing the potential role different mosquito vectors might play in the event this virus is introduced, and informing risk models. Further, a myriad of mosquito species are predicted to contribute to RVFV transmission based on laboratory competency and blood feeding patterns [15], which will necessitate a complex vector surveillance and intervention strategy post-invasion. Therefore, filling in data gaps for species with epidemiologically significant host selection patterns, but for which vector competence data are lacking is paramount.

*Aedes melanimon* Dyar, *Ae*. *vexans* (Meigen), and *Ae*. *dorsalis* (Meigen) were recently shown to feed on both cattle and deer in agricultural northern Colorado plains, suggesting high cross-species transmission risk given adequate vector competence and dispersal [21]. Populations of *Ae*. *vexans* exhibit some geographical variation in their vector competence [18,19], while *Ae*. *dorsalis* from mixed California/Colorado sampling exhibit low vector competence [19]. Vector competence data were previously lacking for *Ae*. *melanimon*. Blood meals from cattle and sheep were also identified in field-collected *Culiseta inornata* (Williston) mosquitoes from northern Colorado. Canadian *Cs*. *inornata* have demonstrated efficient transmission of RVFV (ZH501) previously, as measured by RT-qPCR analysis of saliva samples [22].

To determine the potential for these mosquito species to transmit RVFV between susceptible North American vertebrate hosts, we conducted vector competence experiments with an epidemic, Kenyan strain (Kenya-128B-15) of RVFV. We targeted *Ae*. *melanimon*, *Ae*. *vexans*, and *Ae*. *dorsalis* due to the recently documented blood-host choices in Colorado, and to illuminate their competence for transmitting an epidemic strain of RVFV. A local sampling of *Cs*. *inornata* was included in these experiments to confirm its high susceptibility and transmission efficiency for RVFV Kenya-128B-15. We also included *Aedes increpitus* Dyar based on high abundances in our sampling sites, and *Cx*. *tarsalis* to confirm previously demonstrated high transmission rates, while providing a positive control species. For each of these species, we investigated the progression of virus infection throughout mosquito bodies (midgut infection, dissemination, saliva), as well as potential for vertical transmission of RVFV using infection of ovaries as a proxy. Finally, we developed a within-host model for the functional analysis of infection patterns, as well as the “barriers” to infection [23] for each tissue.

## Methods

### Field collections

**Fig 1:**
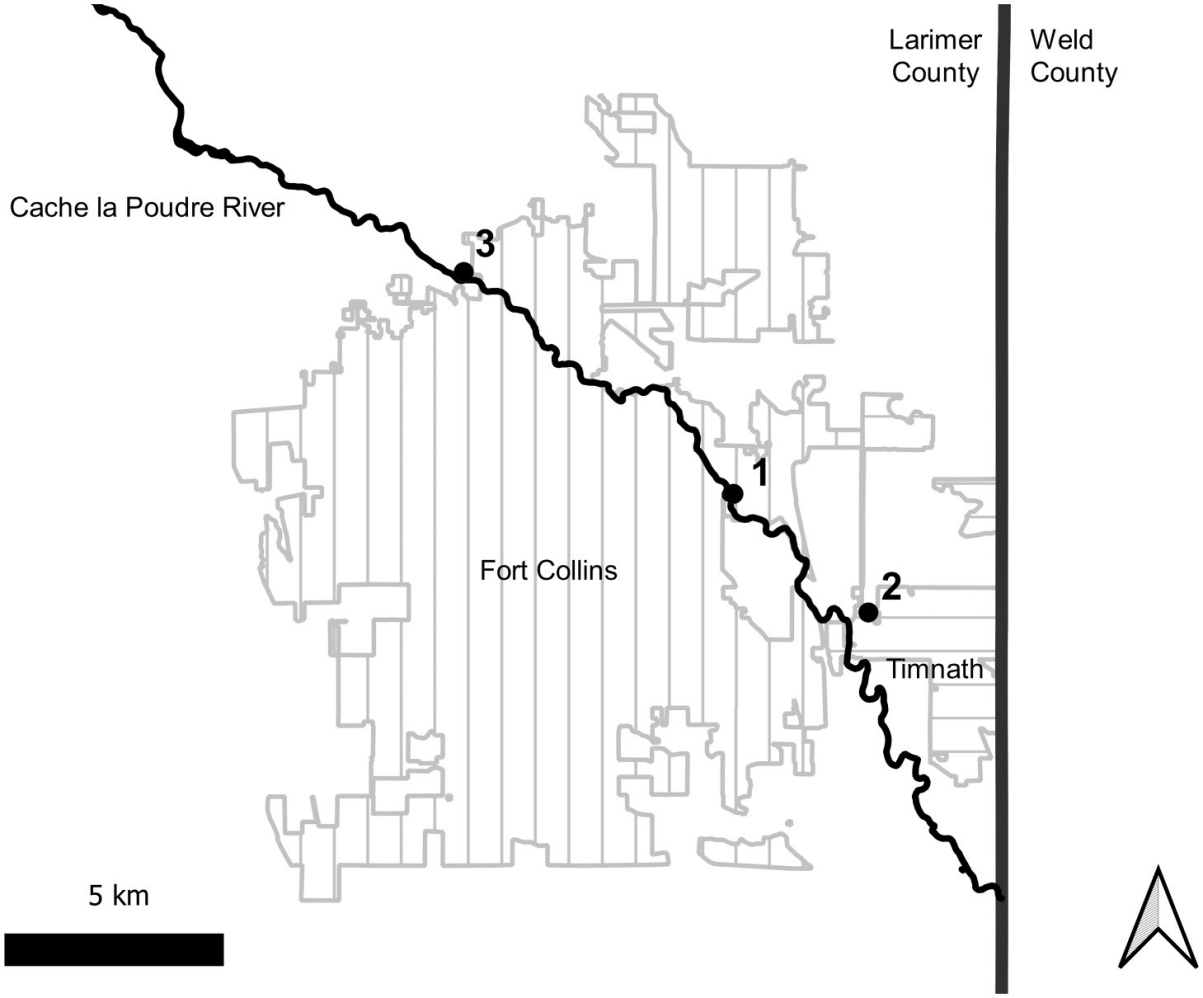
Map of sites for collection of wild mosquitoes in northern Colorado. Numbers indicate the experimental replicate associated with each site. City boundary data available at from Colorado Department of Public Health and Environment (https://data-cdphe.opendata.arcgis.com/datasets/colorado-county-boundaries/) and hydrology data from the United States Geological Survey National Hydrography Dataset https://apps.nationalmap.gov/downloader/.

Field collections of our target species for vector competence experiments were made using CDC light traps, deploying 10 traps per collection effort at three trapping locations (Fig 1). Three replicates of vector competence challenges were completed with field-collected mosquitoes. The first replicate utilized mosquitoes collected from the Environmental Learning Center (N 40.557°, W 105.017) in Fort Collins, Colorado on 6/14/2019. For the second replicate we collected in Timnath, Colorado (N 40.532°, W 104.980°) on 7/3/2019. We collected near the McMurray Natural Area (N 40.603°, W 105.091) in northwestern Fort Collins for the third and final replicate on 7/30/2019.

### Vector competence for RVFV strain Kenya-128B-15

For these studies, RVFV strain Kenya-128B-15 from the 2006–2007 outbreak in Kenya was used [24,25]. This strain was isolated from a pool of *Aedes ochraceus* (Theobald) mosquitoes [25], and passaged four times prior to these experiments (twice on Vero cells, once on C636 cells, and once more on Vero cells). Prior to oral challenge with RVFV, mosquitoes were placed into screened 0.47 L ice cream cartons (Huhtamaki, Espoo, Finland) and acclimated to insectary conditions (26 °C, 70% relative humidity, 16:8 light/dark cycle) for 2–3 days, and were provided with water and sugar cubes *ad libitum*. Mosquitoes were relocated to an incubator in the Biosafety Level 3 laboratory 24 hours prior to virus challenge and deprived of sugar and water.

Virus was prepared for oral challenge by infecting Vero cells (ATCC CCL-81, American Type Culture Collection) at a multiplicity of infection (MOI) of 0.01. Virus was incubated on cell monolayers for one hour at 37 °C, rocking every 15 minutes. On day 3 post-inoculation, virus supernatant was collected and mixed 1:1 with fresh defibrinated calf blood (Colorado Serum Company, Denver, CO), and ATP to a final concentration of 8 mM. This represents the fifth total passage for virus that was used for these challenges. Virus-blood preparation was presented to mosquitoes using a Hemotek Membrane Feeding System (Hemotek, Blackburn, United Kingdom) for 75 minutes, with a small (~ 9g) mass of dry ice near each feeder to encourage feeding by releasing CO_2_. Mosquitoes were cold-immobilized, sorted to separate fully engorged females, and placed in an incubator at 26 °C and 70% relative humidity. One mL of each blood/virus preparation were frozen at -80 °C until titration by plaque assay.

After 14 days of incubation, we identified mosquitoes to the species level using two taxonomic keys [26,27], and harvested saliva, legs/wings, ovaries, and carcasses. We collected saliva as a measure of capacity for horizontal transmission, legs and wings as a measure of viral dissemination, ovaries to determine potential for vertical transmission, and carcasses to determine midgut infection. Mosquitoes were cold immobilized, and legs and wings were removed from each specimen. Saliva was collected by placing the proboscis in the end of a 10 μL capillary tube of Type B immersion oil (Cargille, Cedar Grove, New Jersey) and allowing to expectorate for 30 minutes, after which the end of the capillary tube was placed in 100 μL of mosquito diluent (DMEM supplemented with 10% fetal bovine serum, 1% Penicillin/Streptomycin, 0.1% Gentamycin, and 0.1% Amphotericin B). Finally, ovaries were dissected, and the remaining carcass was collected. All tissues (legs/wings, ovaries, carcasses) were collected in a microcentrifuge tube containing 2 glass Colirollers beads (MilliporeSigma, Burlington, MA) and 200 μL of mosquito diluent. All samples were frozen at -80 °C until analysis.

Mosquito saliva samples were thawed, centrifuged at 11,000 RPM for 5 minutes, diluted serially (1:2–1:2x10^5^) and plaqued on Vero cells. Tissue samples (bodies, legs/wings, ovaries) were thawed, homogenized using a TissueLyser (Qiagen, Hilden, Germany) at 24 Hz for 1 minute, and centrifuged at 14,000 RPM for 1.5 minutes prior to performing plaque assays. Tissue samples were plaqued undiluted, and diluted 1:10–1:10^5^. Plaque assays were performed by plating 125 μL of dilutions of each sample on Vero cell monolayers in 12-well plates in singlicate, and incubated for 1 hour at 37 °C for one hour while rocking the flask every 15 minutes. After incubation, a 2% agarose/DMEM overlay was added. Two days post-inoculation, cells were stained with 0.33% neutral red (Sigma Aldrich, St. Louis, Missouri). Plaques were counted on day 3 post-inoculation. The limit of detection (LOD) for this assay was defined as the corresponding PFU/mL obtained by observing 1 plaque in the least dilute well.

### Confirmation of virus identity

Because wild-caught mosquitoes were used for these experiments, mosquito carcasses were screened by RT-qPCR to confirm the presence of RVFV, and exclude possibility of natural West Nile virus (WNV) detection by plaque assay. RNA extractions were performed using the MagMAX -96 Viral RNA Isolation Kit (Applied Biosystems, Waltham, Massachusetts, United States), and reactions were performed with TaqMan Fast Virus 1-Step Master Mix (Applied Biosystems) using fast cycling parameters on a QuantStudio 3 cycler. The qRT–PCR for RVFV quantification utilized the primers RVFL-2912fwdGG and RVFL-2981revAC at 500 nM final concentration, with probe RVFL-probe-2950 at 100 nM final concentration [28]. West Nile virus assays were performed using the primers WNENV-forward and WNENV-reverse at final concentrations of 500 nM (each), and probe WNENV-probe at a final concentration of 250 nM [29]. RT-qPCR reactions were run in singlicate alongside no-template controls. Standard sets were run in duplicate, utilizing serially diluted RVFV MP12 (vaccine strain) or local (Fort Collins) isolates of WNV. Serial dilutions were plaqued in duplicate according the methods above for relating Ct values to PFU/mL. Default detection thresholds from the Quantstudio 3 software were used.

### Data analysis

To analyze the plaque assay data, we fit a within-host model of mosquito organ infection. Details of the model are included in S1 Appendix and parameters are described in Table 1. This model allowed us to estimate the infection probabilities for each organ, while quantifying the barriers to infection of each organ. Hierarchical Bayesian model structure was devised to gain a measure of uncertainty for these estimates. The model was fit for each species separately using the ‘runjags’ package [30] in the R environment [31]; two parallel Markov Chain Monte Carlo (MCMC) chains were run with 5,000 burn-in iterations and 120,000 monitored samples. Statistical significance between parameter estimates was determined by examining 95% credible intervals (CI’s) for overlap. These analyses assumed that infectious blood meals administered were consistent enough in titer to have negligible effects on the observed infection outcomes.

**Table 1:** Parameters used in within-host model. See also S1 Appendix.

Parameter	Definition	Mathematical Expression
p_1_	Probability of midgut infection	a
p_2_	Probability of ovarian infection	a*b
p_3_	Probability of infection dissemination	a*c
p_4_	Probability of infectious saliva	a*c*d
a	Midgut infection probability	a = p1
b	Probability that established midgut infection spreads to ovaries	a = p2/p1
c	Probability that established midgut infection disseminates to legs and wings	c = p3/p1
d	Probability that disseminated infection produces virus in saliva	d = p4/p3
1-a	Midgut infection barrier	
1-b	Ovarian infection barrier	
1-c	Midgut escape barrier	
1-d	Saliva barrier	

## Results

### Blood meal titers

Infectious blood meals administered to field-collected mosquitoes varied only slightly in titer (Table 2). Mosquito samples from each replicate represent samples for which feeding, incubation, and dissections were completed generating a full sample set (saliva, ovaries, legs/wings, carcasses) (Table 2).

**Table 2:** Numbers of mosquitoes challenged with RVFV Kenya-128B-15 by species and replicate. * Titer for each blood meal administered to mosquitoes.

Species	Replicate 1 ELC *4.0E6 PFU/mL	Replicate 2 Timnath *2.1E6 PFU/mL	Replicate 3 McMurry *7.8E6 PFU/mL	Grand Total
*Aedes increpitus*	0	0	3	3
*Aedes melanimon*	2	29	0	31
*Aedes vexans*	12	27	13	52
*Culex tarsalis*	3	3	15	21
*Culiseta inornata*	2	1	2	5
Grand Total:	19	60	33	112

### Midgut infection

**Fig 2:**
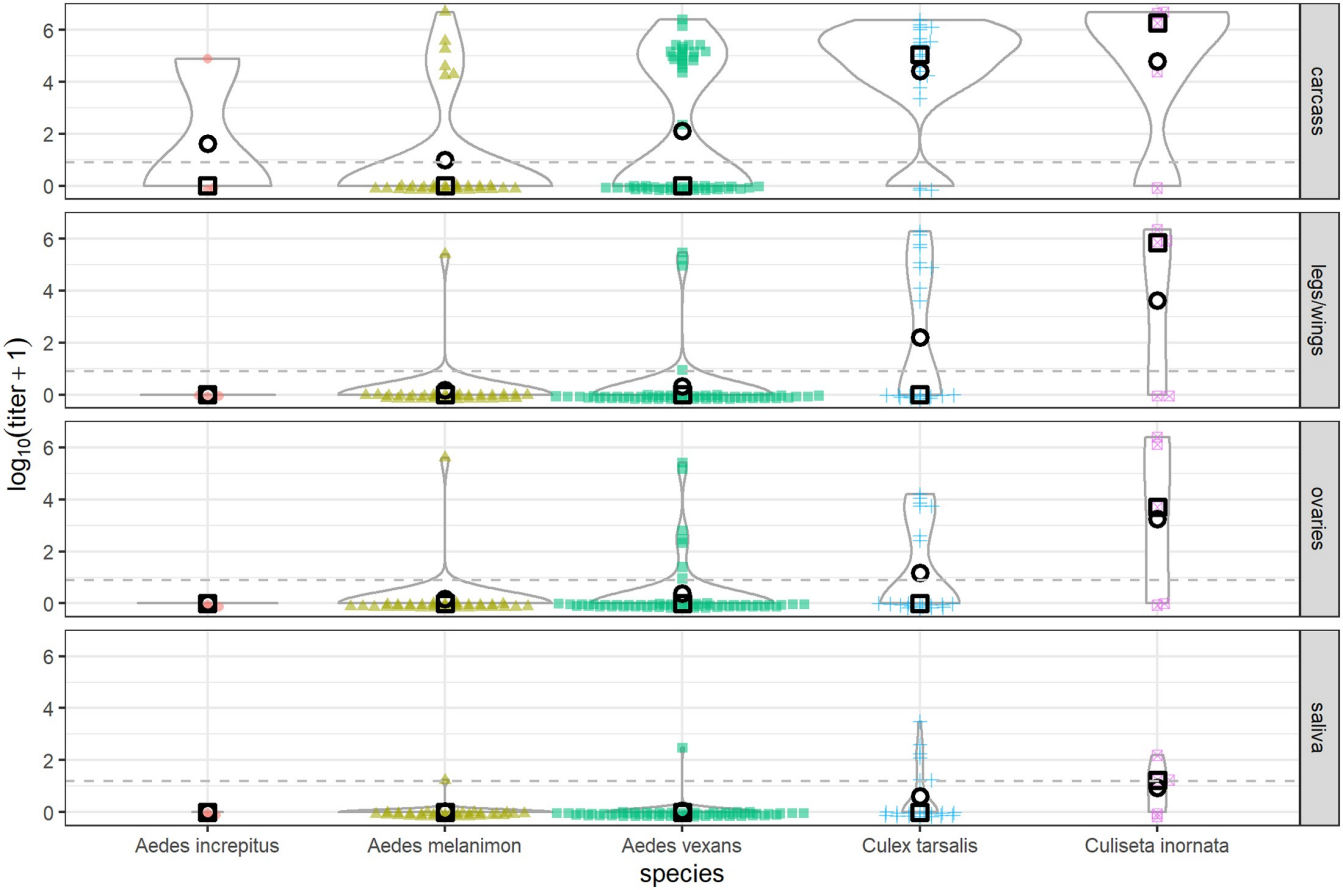
PFU/mL RVFV detected in mosquito tissues by plaque assay. Dashed lines represent limits of detection for the assay. Mean values are shown as open black circles, while median values are shown as open squares. Violin densities show distributions of the data (solid gray lines).

**Fig 3:**
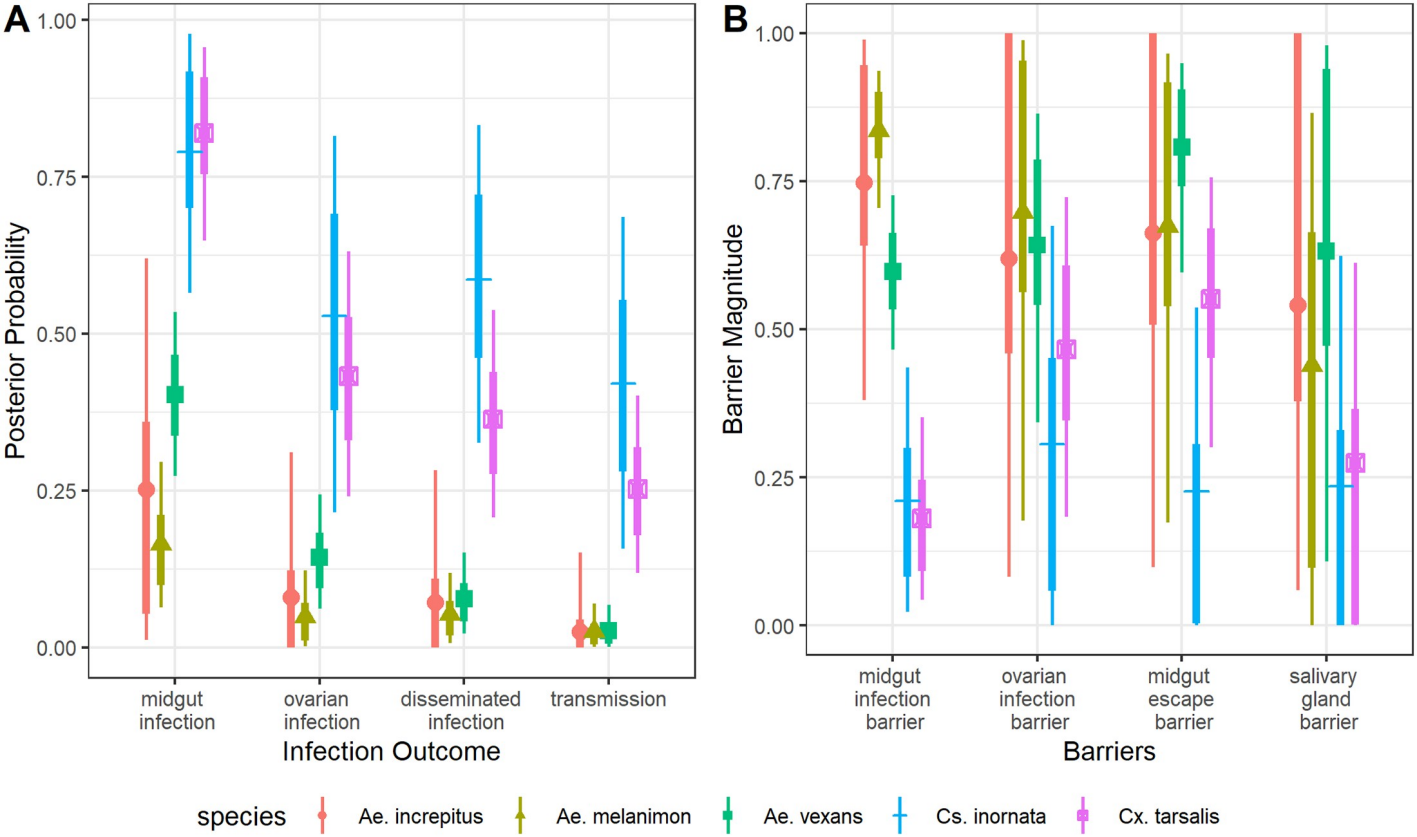
**A** shows the probability of each infection outcome. **B** shows the barriers to infection as independent, step-wise parameters. Points represent median posterior values, while the thin lines represent 95% credible intervals and thick lines represent 66% credible intervals. See also S1 Appendix and Table 1 for the definitions and associated model parameters.

Mosquitoes of each species in this study exhibited viral infections of the midgut, detected by plaque assays of homogenized carcasses (Fig 2). Numbers of positive samples, proportions of positive samples out of total, and 95% CI’s are shown in Table 3. Model estimates for midgut infection probability were significantly higher for *Cx*. *tarsalis* than for all three *Aedes spp*. tested (*Ae*. *vexans*, *Ae*. *increpitus*, and *Ae*. *melanimon*) (Fig 3 and Table 3). Midgut infection probabilities were also significantly higher for *Cs*. *inornata* than *Ae*. *vexans and Ae*. *melanimon* (Fig 3 and Table 3). All RT-qPCR testing confirmed the presence of RVFV RNA in samples with positive plaque assays; none of these samples were positive for WNV by RT-qPCR. Means, medians, 95% credible intervals, and standard deviations to describing the posterior distributions are included as S1 Table, and posterior distributions are shown in S1 Appendix.

**Table 3:** Sample sizes and positive samples by species and tissue type. Numbers of positive sampled are expresses as a total number, followed by (proportion positive, 95% CI range). Range of 95% CI are given from estimates of p1, p3, p2, p4 for carcasses, legs/wings, ovaries, and saliva, respectively.

Species	Sample Size	carcass	legs/wings	ovaries	saliva
*Aedes increpitus*	3	1 (0.33, 0.02–0.62)	0 (0, 0.00–0.28)	0 (0, 0.00–0.31)	0 (0.00–0.15)
*Aedes melanimon*	31	6 (0.19, 0.06–0.30)	1 (0.03, 0.01–0.12)	1 (0.03, 0.00–0.12)	1 (0.03, 0.00–0.07)
*Aedes vexans*	52	22 (0.42, 0.27–0.53)	3 (0.06, 0.02–0.15)	6 (0.12, 0.06–0.24)	1 (0.02, 0.00–0.07)
*Culex tarsalis*	21	18 (0.86, 0.65–0.96)	9 (0.43, 0.21–0.54)	7 (0.33, 0.24–0.63)	6 (0.29, 0.12–0.40)
*Culiseta inornata*	5	4 (0.80, 0.56–0.98)	3 (0.60, 0.33–0.84)	3 (0.60, 0.21–0.81)	3 (0.60, 0.16–0.69)

### Viral dissemination

Viral dissemination from the midgut of the mosquito, indicated by virus detection in legs and wings of mosquitoes, occurred in all species with the exception of *Ae*. *increpitus* (Fig 2 and Table 3). There were significantly higher model estimates for probability of dissemination among *Cx tarsalis* than both *Ae*. *melanimon* and *Ae*. *vexans* (Fig 3A and Table 3). *Culiseta inornata* also had a higher probability of having disseminated infections than all three *Aedes spp*. tested (Fig 3A and Table 3). *Cs*. *inornata* also showed a significantly lower midgut escape barrier than *Ae*. *vexans* (Fig 3B and S1 Table).

### Transmission via saliva

Infectious virions were detected in the saliva of at least one individual mosquito representing every mosquito species tested in these experiments except for *Ae*. *increpitus* (Fig 2 and Table 3). Transmission was assumed for mosquitoes with any measurable virus in saliva by plaque assay, and parameter estimates for p4 (S1 Appendix) were interpreted as the probability of transmission. Median transmission probability for *Cs*. *inornata* was the highest followed by *Cx*. *tarsalis*, then *Ae*. *vexans*, *Ae*. *melanimon*, and *Ae*. *increpitus* (Fig 3A). Transmission probability was significantly higher for *Cs*. *inornata* than for all three *Aedes spp*. tested, and also significantly higher for *Cx*. *tarsalis* than both *Ae*. *melanimon* and *Ae*. *vexans* (Fig 3A and Table 3). Though we did not detect virus in saliva samples from *Ae*. *increpitus*, sample sizes were low for this species (n = 3); therefore, the possibility of transmission by this species cannot be eliminated.

### Ovarian infection

Finally, ovaries were tested by plaque assay in order to investigate the possibility of vertical transmission. Numbers of infected ovary samples are shown in Table 3. *Cs*. *inornata* showed the highest median probability of ovarian infection, followed by *Cx*. *tarsalis*, *Ae*. *vexans*, *Ae*. *increpitus*, and *Ae*. *melanimon* (Fig 3A). Probability of ovarian infection was significantly higher for *Cx*. *tarsalis* than both *Ae*. *melanimon* and *Ae*. *vexans* (See S1 Table for unrounded 95% CI’s regarding *Cx tarsalis* and *Ae*. *vexans* comparisons; this difference is marginal). Ovarian infection probability was also higher for *Cs*. *inornata* than *Ae*. *melanimon*. Interestingly, we observed four *Ae*. *vexans* mosquitoes for which ovaries tested positive, while corresponding legs/wings were not (Fig 2 and Table 3).

## Discussion

This study assessed the ability of wild-caught mosquitoes from Colorado to become infected with and transmit an outbreak strain of RVFV. Among the species assessed are those that are documented to feed upon potential local amplifying hosts of RVFV, and two mosquito species for which vector competence had not yet been assessed (*Ae*. *melanimon* and *Ae*. *increpitus*). This study also represents the first set of experiments to test several North American mosquitoes for a more recent epidemic strain than that historically used. The data presented here confirm the ability of several of these mosquito species, all with host breadths including RVFV-susceptible vertebrate hosts, to transmit RVFV by bite. Incubation temperatures and durations were chosen for consistency with previous work [17–20] and environmental conditions in northern Colorado. In order to understand infection patterns at the organismal level, several tissues were harvested from mosquitoes and tested for infectious virions by plaque assay. While sample sizes are relatively low for *Cs*. *inornata* and *Ae*. *increpitus*, we were able to draw credibility intervals on the susceptibilities of these mosquitoes by using a within-host model (Fig 3). The novel within-host model allowed us to assess the relative importance of infection and transmission barriers in different species, although additional data would be useful for *Ae*. *increpitus* and *Cs*. *inornata* to provide useful parameter estimates that can be used for informing control and model parameterization.

### Blood meal titers

Viral titers encountered by naïve mosquitoes can vary widely depending on the host species, host age, and period of viremia. The viral titers in the blood meals administered to mosquitoes were realistic representations of peak viremias reached in 4-5-month-old North American Polypay sheep [32], 5-month-old white-tailed deer [16], and 7-day-old calves [33], all of which developed peak titers between 6–8 log_10_ PFU/mL so our inoculum may represent either transient or peak viremias of these animals (Table 2). Due to the nature of RVFV blood viremias in these vertebrate hosts, the blood meal titers administered in these experiments likely represent the higher end of the spectrum. The viremias that may develop in domestic North American cattle are not well investigated at the time of this writing; this should be a research priority given the relationship between viremia and mosquito susceptibility [15].

While we were intentional in providing mosquitoes an artificial blood meal containing freshly cultured virus, our data may underestimate true infection, dissemination, and transmission rates that would be observed in nature, where mosquitoes would be feeding on viremic animals. Infection rates have been shown to be considerably higher in mosquitoes feeding on a viremic host as compared with those exposed to an artificial infectious blood meal [34–36]. Further, mosquitoes fed on lambs viremic for RVFV had a higher engorgement rate and higher rate of saliva-positive individuals than mosquitoes exposed to RVFV through a membrane feeder [37].

### Midgut infection probability

Infection probabilities for *Ae*. *vexans* in this study were not markedly different from previously reported infections using mixed Colorado/California *Ae*. *vexans* populations [19], and still below infection probabilities for the moderately competent *Ae*. *vexans* population from Florida [18]. Population-level variation in susceptibility of *Ae*. *vexans* to infection may reflect genetic factors [38], or variation among experimental methods. Infection probabilities of *Ae*. *increpitus* and *Ae*. *melanimon* were moderate, and did not differ significantly from *Ae*. *vexans* in this study. Midgut infection probabilities were relatively high for the permanent water breeders *Cx*. *tarsalis* and *Cs*. *inornata* (Figs 2 and 3). Previous infection rates of *Cx*. *tarsalis* exposed to a higher dose (7.3 log_10_ PFU/mL) were also high [19], consistent with observations in this study. Infection probabilities for *Cs*. *inornata* were also high, similar to previous experiments using *Cs*. *inornata* mosquitoes from Canada [22].

### Dissemination probabilities

Viral dissemination from the midgut, or midgut escape, requires virus particles to pass through the basal lamina of the mosquito gut into the hemolymph. As with midgut infection, disseminated infection probabilities (Fig 3A) were low for *Aedes vexans*, consistent with those previously demonstrated with *Ae*. *vexans* from mixed Colorado/California collections [19]. This previous work demonstrated strong midgut infection barriers, as well as strong midgut escape barriers in *Ae*. *vexans*, resulting in overall low transmission efficiency. Data presented here support this observation; for *Ae*. *vexans*, the midgut escape barrier was the highest estimated (Fig 3B). Disseminated infection probabilities were similar for the other floodwater species, *Ae*. *increpitus* and *Ae*. *melanimon* (Fig 3A). Dissemination also trended higher for *Cx*. *tarsalis* and *Cs*. *inornata* compared to the *Aedes spp*. *Cs*. *inornata* exhibited an especially low midgut escape barrier (Fig 3B), in addition to its low midgut infection barrier.

### Transmission probabilities

Transmission, defined as detectable virus in saliva, trended higher for *Cx*. *tarsalis* and *Cs*. *inornata* than the *Aedes spp*. (Fig 3A). Previous transmission efficiency data for *Cx*. *tarsalis*, which used infected and susceptible hamsters to test for transmission were high similar to data reported here [19]. The transmission probability estimated for *Ae*. *vexans* and *Ae*. *melanimon* were low as demonstrated previously [19]. However, given the high abundances for these species in Colorado, as well as their blood-host preferences for susceptible vertebrate hosts [21], they might contribute significantly to RVFV transmission. The only species we tested that did not show positive saliva for RVFV was *Ae*. *increpitus*; however, the sample size was small, and Bayesian estimation of transmission probability for these mosquitoes yielded 95% CI’s similar to the other floodwater species, so transmission cannot be ruled out entirely. *Ae*. *increpitus* mosquitoes have exhibited blood-host preferences including a large proportion of mule deer [39], and may still make a contribution to RVFV maintenance in the United States.

The results from some individuals that were positive but directly on the limit of detection of the plaque assay are difficult to interpret in terms of biological relevance. This was evident for some saliva samples (Fig 2) from *Cs*. *inornata*, *Cx*. *tarsalis* and *Ae*. *melanimon*, all of which had a disseminated infection. It also occurred more often for sets of samples that had relatively lower titers (5 salivas, 1 ovaries, 1 legs/wings, 0 carcasses). Removing these individuals from the data as positives and running the model produced slightly different parameter estimates (S2 Appendix) but does not qualitatively change the conclusions made here. While low RVFV titers have been reported elsewhere [6,40,41], contamination cannot be ruled out entirely.

### Ovarian infection

RVFV also presents some ecological complexity due to its ability to be vertically transmitted by mosquitoes. There is strong evidence for vertical transmission among *Ae*. *macintoshi* (previously referred to as *Ae*. *lineatopennis*) mosquitoes from Kenya, contributing to viral maintenance through inter-epidemic periods [3]. Proportions of mosquito ovaries with detectable virus may not relate directly to the proportion of infected progeny. Additionally, transovum transmission may occur prior to transovarial transmission. However, these data provide preliminary evidence that vertical transmission may be possible in these mosquito species. Again, there was a trend toward higher ovarian infections probabilities in *Cx*. *tarsalis* and *Cs*. *inornata* compared to *Ae*. *vexans*, *Ae*. *melanimon*, and *Ae*. *increpitus* (Fig 3A). Though we do not see statistically significant differences among many of the internal infection barrier estimates (Fig 3B), median ovarian infection barrier estimates were lower for *Cx*. *tarsalis* and *Cs*. *inornata*, suggesting that this is not all attributable to differences in midgut infection and escape barriers.

We made an interesting observation with four *Ae*. *vexans* mosquitoes, for which ovaries were positive for infectious virus in the absence of viral dissemination from the midgut. This observation has been made with experimentally infected *Cx*. *tarsalis* mosquitoes [6], but it cannot be ruled out that some disseminated infections were missed due to low viral loads in legs/wings relative to the limit of detection. The titers of positive ovaries were low (approximately 8, 24, 629, and 292 pfu/mL tissue sample), so it is also possible that these were false positives. By considering these samples to be negative, the median ovarian infection probability for *Ae*. *vexans* is reduced from 0.14 to 0.05 (S1 Table, S2 Appendix). Similar patterns have been observed with La Crosse virus in the vector *Aedes triseriatus* (Say) [42]. RVFV has been detected in the tracheal system of mosquitoes, and this has been hypothesized as an alternative route of dissemination to classical midgut escape in which virus passes through the gut and basal lamina [4,43–44]. This route of ovarian infection is recognized for other mosquito-borne viruses [45–46]. This phenomenon we observed would be best confirmed using confocal imaging techniques rather than plaque assay as conducted in these experiments. Independence between these routes of infection is accounted for in our model structure.

## Conclusion

Collectively, these results reinforce the hypothesis that transmission of RVFV among various wildlife species and domestic ungulates in the United States would likely involve several mosquito vector species [15]. This complexity presents a major challenge for the implementation of vector surveillance and control strategies in the event of an invasion of RVFV. The detection of infectious virus in mosquito ovaries in several of these species is especially troubling. Vertical transmission by *Aedes spp*. would result in additional viral reservoirs in desiccation-resistant egg populations, while vertical transmission by *Culex* and *Culiseta*, which overwinter as adults, would enhance early season amplification in temperate zones where these mosquitoes diapause. While we observed interesting trends between mosquito species with these different oviposition strategies, intra- and interspecific genetic differences among mosquito populations were not taken into consideration in this study and would also influence differences in virus susceptibility. Further studies should investigate the viral tropism in F1 generation mosquitoes to determine any transstadial barriers that may or may not exist.

We developed a within-host model for the analysis of vector competence data. This offers many advantages over qualitative descriptions. First, this model offers mathematical definitions that formalize ideas such as infection barriers. Fitting this model to data offers a holistic, functional analysis to estimate these parameters while producing measures of uncertainty (95% CI’s). This is especially useful for studies using wild-caught mosquitoes and select agent pathogens, wherein sample sizes can be small due to poor feeding success or survivorship under laboratory conditions. However, with small sample sizes, parameter estimates should be interpreted with caution. For *Ae*. *increpitus* and *Cs*. *inornata* in these experiments, these model estimates have value as prior distributions for further work rather than accurate estimates to inform transmission dynamics. Finally, this model can be easily extended to include any number of covariates, such as blood meal titer and incubation temperature. We recommend the use of such models for future vector competence work, so that rigorous comparisons can be made between experiments.

## Supporting information

S1 Appendix Description of within-host model of arbovirus infection. (PDF)

S2 Appendix Two alternative analyses are presented here. First, we raise the LOD to require at least two plaques in the least dilute well of the 12-well plate to count samples as positive for RVFV. Second, ovary samples with detectable RVFV from *Ae*. *vexans* mosquitoes with non-disseminated infections are counted negative to explore the contribution of these samples on the estimated ovarian infection probability and ovarian infection barrier.
(PDF)

S1 Table Spreadsheet provides descriptions for the posterior distributions of the parameters estimated using the model. See Table 1 and S1 Appendix for definitions of these parameters. The spreadsheet provides the 95% credible intervals (Lower95 and Upper95), Medians, Means, and standard deviations (SD’s).
(XLSX)
