# Supplementary material for: Susceptibility and barriers to infection of Colorado mosquitoes with Rift Valley fever virus
Source: PLoS Negl Trop Dis. 2021 Oct 25;15(10):e0009837. doi: 10.1371/journal.pntd.0009837 (PMC8568276; doi:10.1371/journal.pntd.0009837)
Supplement: S1 Appendix — (PDF) [file pntd.0009837.s001.pdf]

Within-Vector Model of Arbovirus Infection

Daniel Hartman

Purpose

Vector competence is generally defined as the ability of an arthropod vector to transmit a pathogen. For arboviruses which rely on propagative transmission, it is of interest to describe any barriers, or bottlenecks that limit the progression of infection throughout the organs of the vector. These infection barriers ultimately limit the ability of the arthropod to transmit the virus. For example, a strong barrier to infection of the midgut, in turn, limits the ability of the virus to disseminate to the salivary glands. The purpose of this model application is to 1. formalize theory for analyzing infection of mosquitoes with arboviruses, 2. estimate the infection rates of various tissues from vector competence data, and 3. form a base model for incorporating covariates of interest, such as temperature, blood meal titer, and virus strain.

Conceptual Model

Figure 1 describes the progression of arbovirus infection in a competent mosquito vector as a sequence of events. First, after ingesting a blood meal from a viremic host, the virus establishes a midgut infection (Figure 1, "a"). After replicating in midgut cells, the virus then passes through the midgut epithelium into the circulating hemolymph ("c"). Infection of the salivary glands then takes place. With saliva now containing infectious virions, the mosquito can expectorate virus into a new vertebrate host ("d"). For some arboviruses such as Rift Valley Fever Virus, virus can be transmitted to offspring through the reproductive system. While this may be dependent upon midgut infection, it represents a second path of infection through the tracheal system to the ovaries ("b"), that is independent of viral dissemination through the circulatory system.

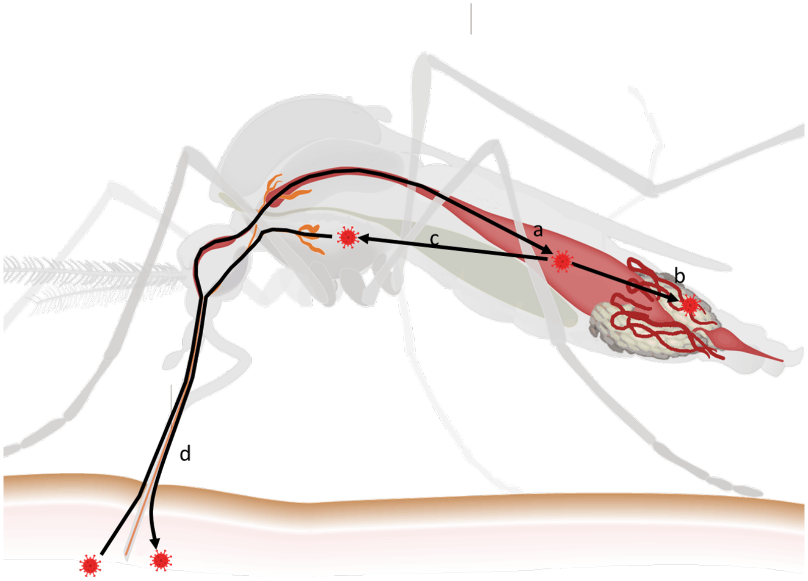

Figure 1: Conceptual Model of Arbovirus Infection of a Mosquito Vector. Each letter can be thought of to represent the probability at which the virus is transmitted *between* organs. Figure modified from the original by Dr. W. Augustine Dunn; Oxitec; The Anatomical Life of the Mosquito, R. E. Snodgrass

The Model

We have constructed a simple Bayesian Model to estimate susceptibility to infection of mosquito organs collected throughout a vector competence experiment, in which native Colorado mosquitoes were assessed for the competence for Rift Valley Fever Virus (RVFV). This model was constructed based on our sample collection methods, where dissections were performed to collect saliva, legs/wings (as an indicator for dissemination), ovaries (indicating potential for vertical transmission), and remaining carcass (as an indicator for midgut infection). The following can be modified for more detailed follow-up sample collection, for example, where salivary glands are removed to tease apart salivary gland infection and salivary gland escape barriers.

Likelihood

Infection status of each mosquito organ follows a Bernoulli distribution. $\mathbf{m}$ is the data vector of mosquito midguts, where $\mathbf{m}_i = 1$ for an infected midgut from individual i, and $\mathbf{m}_i = 0$ for an uninfected midgut. $\mathbf{o}$ is the ovary data vector, $\mathbf{d}$ is the vector of leg/wing data and $\mathbf{s}$ is the vector for the saliva data.

$$\mathbf{m}_i \sim Bernoulli(p_1)$$
$$\mathbf{o}_i \sim Bernoulli(p_2)$$
$$\mathbf{d}_i \sim Bernoulli(p_3)$$
$$\mathbf{s}_i \sim Bernoulli(p_4)$$

As p_1 is the probability of a mosquito acquiring a midgut infection, it is equal to a (Figure 1). p_2, the probability of ovarian infection in a mosquito following a bloodmeal, however is the result of midgut infection establishment *and* spread to the ovaries. In this case it is the product of the probabilities of those two events, or $a \times b$. The probability of a disseminated infection, p_3 is $a \times c$, and the probability of virus in saliva (transmission, p_4) is $a \times c \times d$.

$$p_1 = a$$
$$p_2 = a \times b$$
$$p_3 = a \times c$$
$$p_4 = a \times c \times d$$

Priors

Uninfomative prior distributions were chosen to have a minimal effect on posterior distributions for each p.

$$p_i \sim beta(1, 1)$$

Barrier Definitions

It is intuitive to think of the midgut infection barrier as the probability that infection does not occur. We can then define the midgut infection barrier as $1 - a$, the ovarian infection barrier as $1 - b$, the midgut escape (dissemination) barrier as $1 - c$, and the salivary gland barrier as $1 - d$. Note that the salivary gland barrier estimated from our data represents a combination of the salivary gland infection and escape barriers.

The Data

Infectious virus particles were quantified in each sample by plaque assay, and expressed in terms of PFU/mL. Squares in the plot represent median titer values for each tissue for each species, while open circles represent mean values.

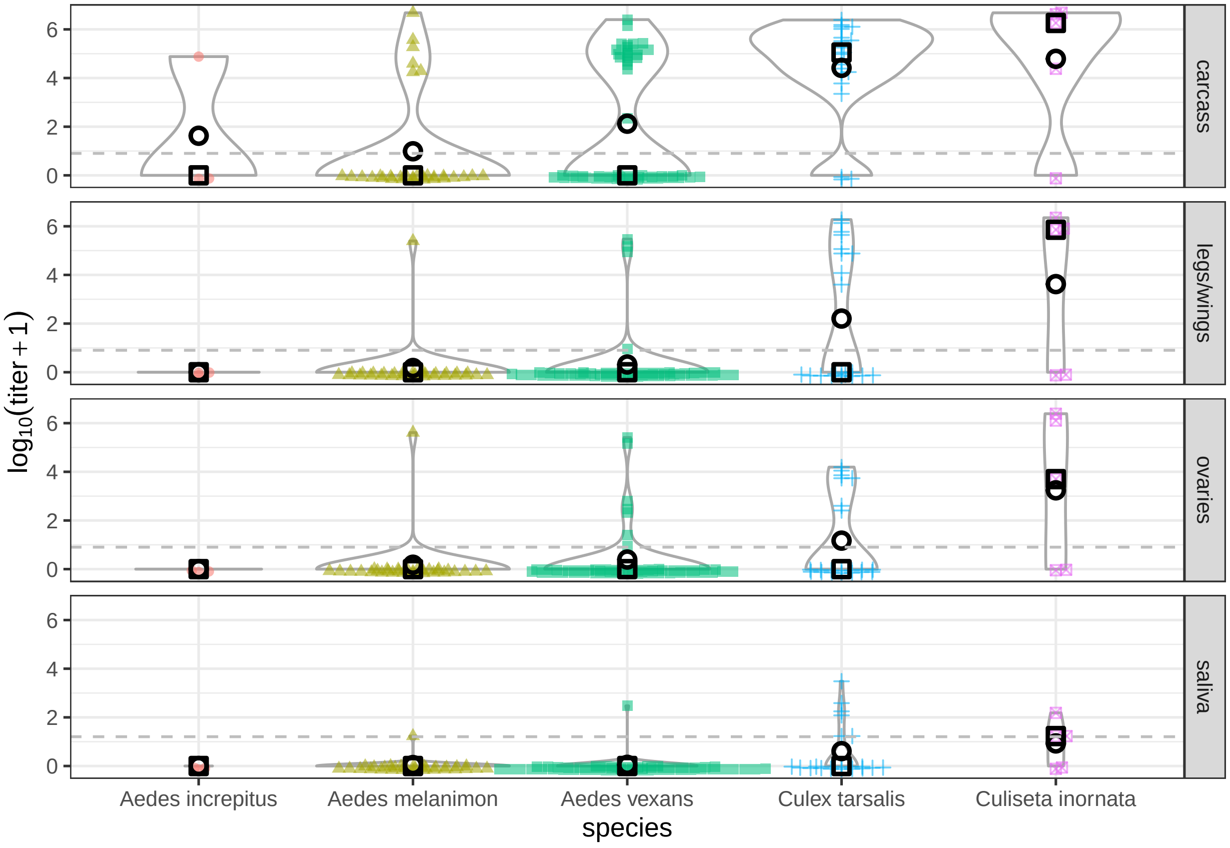

For model fitting, all positive samples were assigned 1, and negative samples assigned 0.

Model Implementation:

JAGS model:

```
model <- "model{
  # Priors
a ~ dbeta(1, 1)
b ~ dbeta(1, 1)
c ~ dbeta(1, 1)
d ~ dbeta(1, 1)

# The data model
for(i in 1:length(M) ) {
  M[i] ~ dbern(a)
}
for(i in 1:length(O) ) {
  O[i] ~ dbern(a*b)
}
for(i in 1:length(D) ) {
  D[i] ~ dbern(a*c)
}
for(i in 1:length(S) ) {
  S[i] ~ dbern(a*c*d)
}
p1=a
p2=a*b
p3=a*c
p4=a*c*d
}"
```

Model Fitting

The model was fit using the ‘runjags’ package, using 5000 burn-in iterations and 120,000 monitored iterations:

```
fit_vex<-run.jags(data=data_list_vex, model=model,
                  monitor=c("a", "b", "c", "d", "p1", "p2", "p3", "p4"),
                          burnin = 5000, sample=120000)
```

convergence Checks

Convergence was verified by inspection of traceplots:

```
## Generating plots...
```

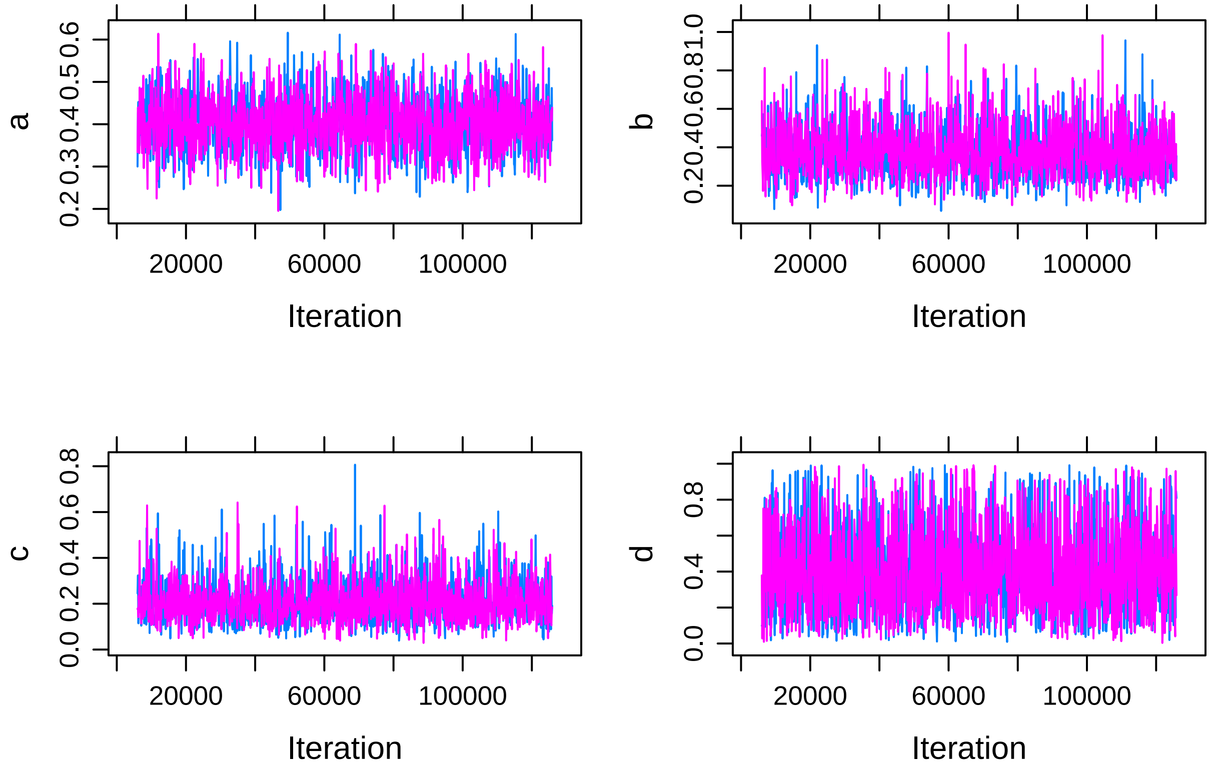

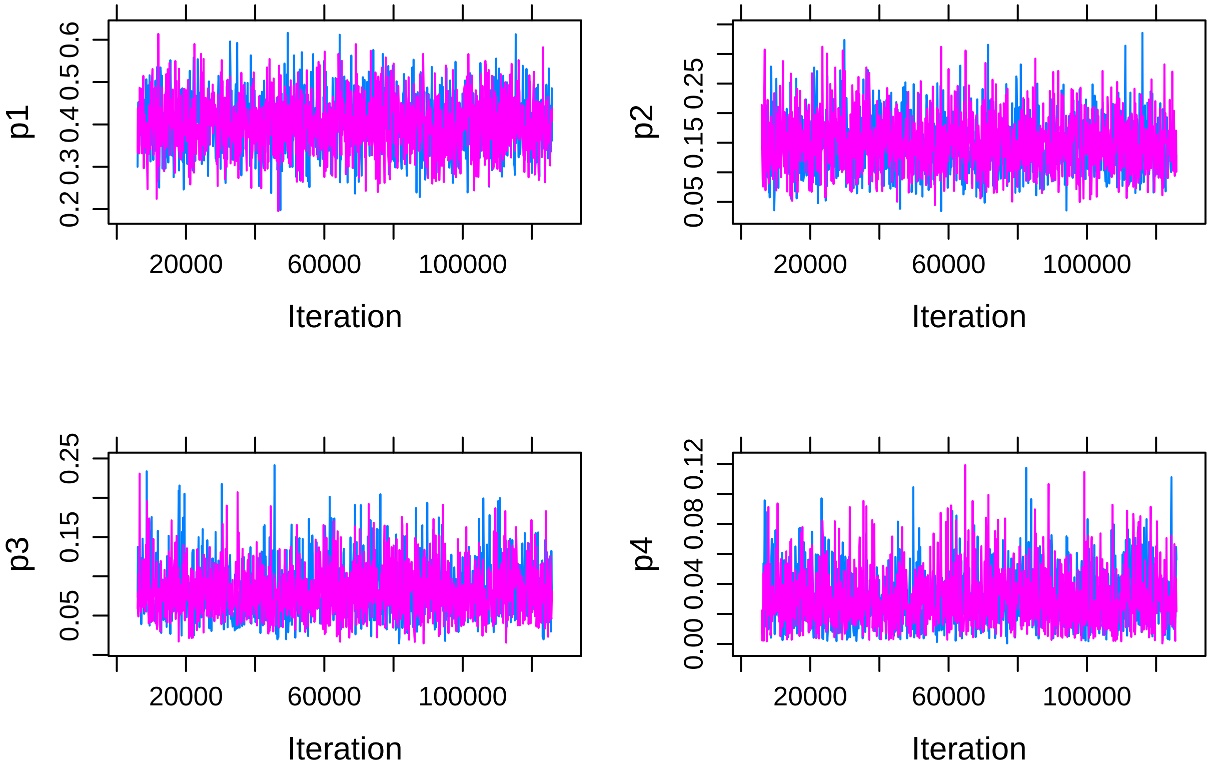
p1
0.2 0.3 0.4 0.5 0.6
20000 60000 100000
Iteration
p2
0.05 0.15 0.25
20000 60000 100000
Iteration
p3
0.05 0.15 0.25
20000 60000 100000
Iteration
p4
0.00 0.04 0.08 0.12
20000 60000 100000
Iteration

Results:

Infection Outcomes

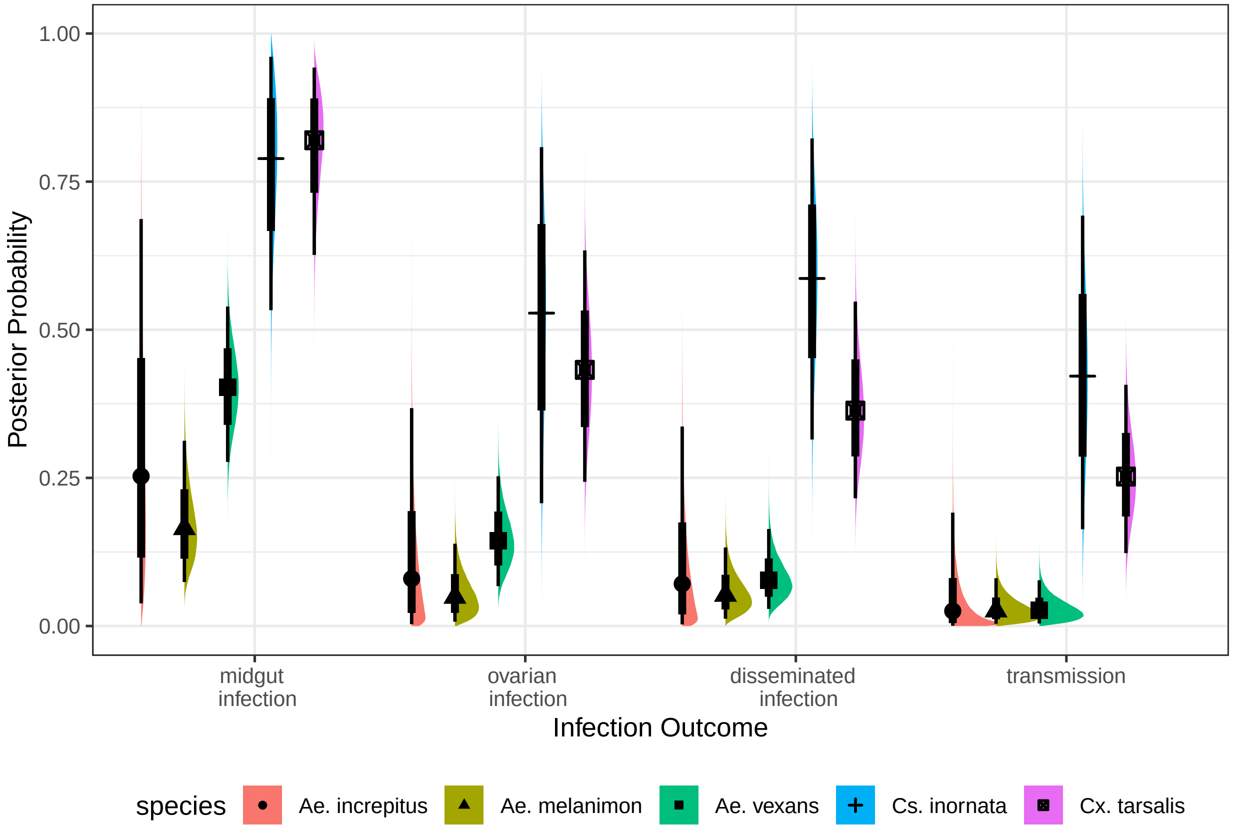

The plot above shows the infection outcomes (p_1 - midgut-limited infection; $p2$ - midgut infection spread to ovaries; $p3$ - disseminated midgut infection; $p4$ - infection and transmission via saliva). Posterior densities are shown along ith the 95% credible intervals (thin lines) and 66% credible intervals (thick lines).

Finally, plotting the barriers (1-a, 1-b, etc.):

Infection Barriers

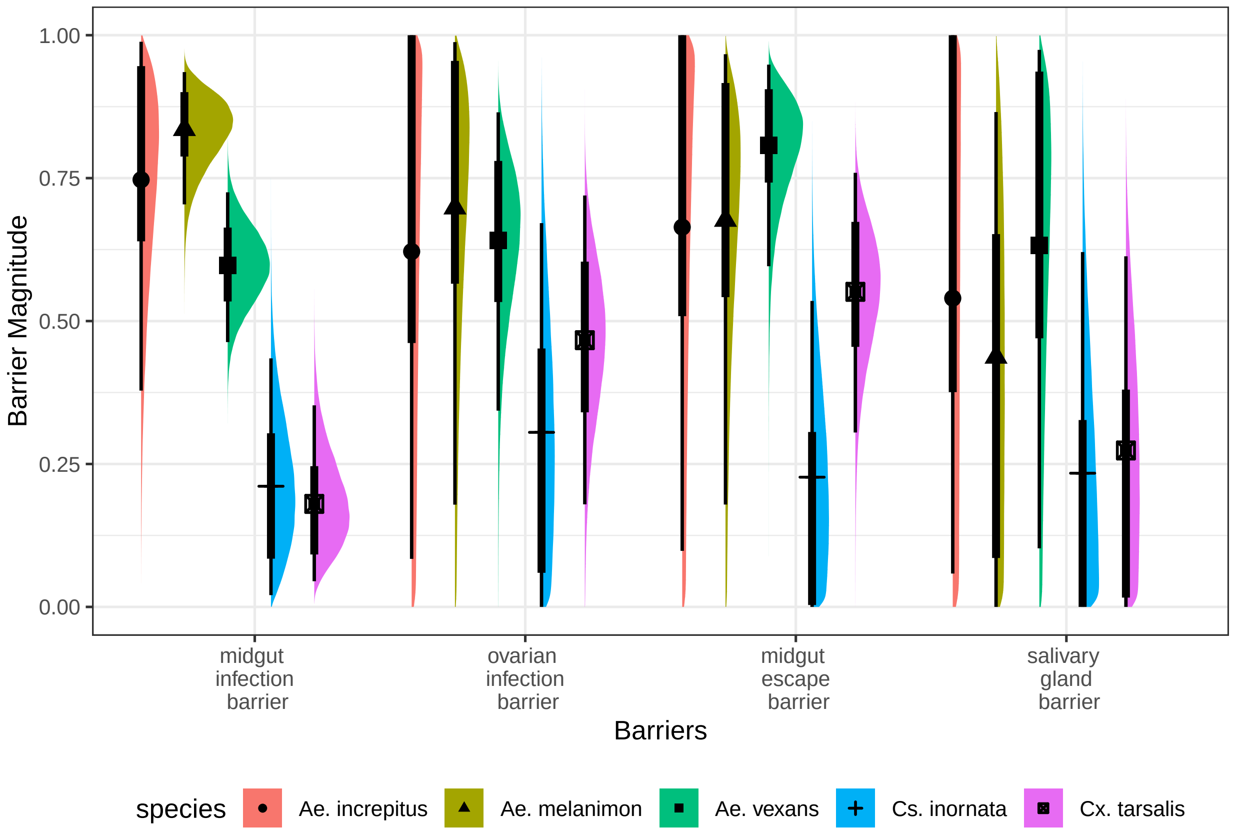

The above plot shows the individual, stepwise barriers to infection ($1-a$ - midgut infection barrier; $1-b$ - ovarian infection barrier; $1-c$ - midgut escape barrier; $1-d$ - salivary gland barrier). Posterior densities are shown along ith the 95% credible intervals (thin lines) and 66% credible intervals (thick lines).
