# Supplementary material for: Susceptibility and barriers to infection of Colorado mosquitoes with Rift Valley fever virus
Source: PLoS Negl Trop Dis. 2021 Oct 25;15(10):e0009837. doi: 10.1371/journal.pntd.0009837 (PMC8568276; doi:10.1371/journal.pntd.0009837)
Supplement: S2 Appendix — First, we raise the LOD to require at least two plaques in the least dilute well of the 12-well plate to count samples as positive for RVFV. Second, ovary samples with detectable RVFV from Ae. vexans mosquitoes with non-disseminated infections are counted negative to explore the contribution of these samples on the estimated ovarian infection probability and ovarian infection barrier. (PDF) [file pntd.0009837.s002.pdf]

S2 - Alternative Analyses

Alternative analysis #1

Alternative analyses are presented here. First, imposing a stricter limit of detection (LOD) on the samples such that data representing only a single plaque in the least dilute well are negative. These data are difficult to interpret biologically, as they are very low (directly on the LOD).

The dashed line in this plot represents these increased (doubled) LODs:

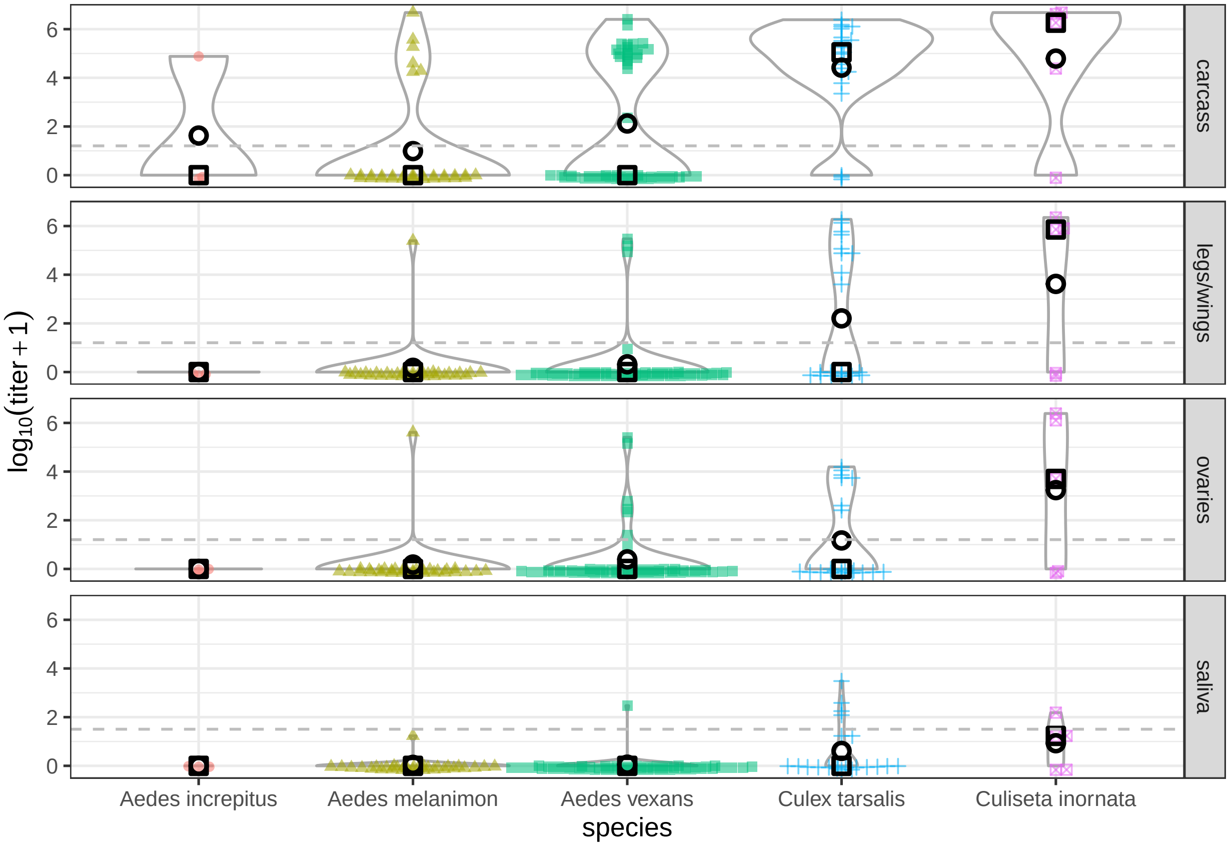

Parameter Summaries

Ae. increpitus

```
##          Lower95     Median   Upper95       Mean         SD
## a  8.568978e-03 0.25151252 0.6128900 0.28220852 0.17182704
## b  7.307390e-05 0.37495616 0.9134343 0.41478461 0.28123709
## c  4.749630e-05 0.33497978 0.9006250 0.38728351 0.27693190
## d  5.754296e-06 0.45719054 0.9411043 0.47215216 0.28742333
## p1 8.568978e-03 0.25151252 0.6128900 0.28220852 0.17182704
## p2 1.206423e-05 0.07981481 0.3056330 0.10788705 0.09872552
## p3 3.678876e-06 0.06992794 0.2840793 0.09764256 0.09112162
## p4 5.049587e-07 0.02528381 0.1506052 0.04366350 0.05213999
```

Ae. melanimon

```
##          Lower95     Median    Upper95       Mean         SD
## a  5.945253e-02 0.16340901 0.29376967 0.17065951 0.06288187
## b  1.547947e-02 0.30313806 0.81966070 0.35300661 0.22946007
## c  5.639283e-03 0.23068449 0.73010766 0.28646237 0.21000761
## d  6.629223e-05 0.34525893 0.90565229 0.39342874 0.27964729
## p1 5.945253e-02 0.16340901 0.29376967 0.17065951 0.06288187
## p2 2.252405e-03 0.04837810 0.12212296 0.05477619 0.03472550
## p3 1.562680e-03 0.03716880 0.10194367 0.04359545 0.02961998
## p4 1.863820e-07 0.01066985 0.04445069 0.01520965 0.01476808
```

Ae. vexans

```
##          Lower95     Median    Upper95       Mean         SD
## a  0.2745370239 0.40301614 0.54047127 0.40440908 0.06760163
## b  0.1078152948 0.31067990 0.59030542 0.32904088 0.12983050
## c  0.0302885769 0.15200371 0.33578324 0.16826991 0.08581519
## d  0.0338149437 0.40982214 0.92524314 0.44500257 0.25666936
## p1 0.2745370239 0.40301614 0.54047127 0.40440908 0.06760163
## p2 0.0464632590 0.12445583 0.21672608 0.12931511 0.04530692
## p3 0.0144263685 0.06074643 0.12864432 0.06608122 0.03117923
## p4 0.0009048742 0.02329021 0.05953328 0.02656256 0.01711610
```

Culiseta inornata

```
##        Lower95    Median   Upper95      Mean        SD
## a  0.52120007 0.7667725 0.9767248 0.7550092 0.1242620
## b  0.33613169 0.7033554 0.9999925 0.6852902 0.1942629
## c  0.33264589 0.6816380 0.9999853 0.6692886 0.1931299
## d  0.09533310 0.4822852 0.9748102 0.4962584 0.2476051
## p1 0.52120007 0.7667725 0.9767248 0.7550092 0.1242620
## p2 0.20532267 0.5155567 0.7999907 0.5124882 0.1566899
## p3 0.20979731 0.5006344 0.7852586 0.4989929 0.1506297
## p4 0.01750849 0.2220409 0.4794544 0.2377066 0.1287684
```

Culex tarsalis

```
##       Lower95    Median   Upper95      Mean         SD
## a  0.64999196 0.8195297 0.9545481 0.8106020 0.08173454
## b  0.27650921 0.5337625 0.8191215 0.5408324 0.13865900
## c  0.18151818 0.3962228 0.6542838 0.4087256 0.12426398
## d  0.13184720 0.4570871 0.9013115 0.4830186 0.20722354
## p1 0.64999196 0.8195297 0.9545481 0.8106020 0.08173454
## p2 0.23702783 0.4330565 0.6301284 0.4337795 0.10128395
## p3 0.15204575 0.3209948 0.5047344 0.3275933 0.09190127
## p4 0.04260202 0.1432990 0.2587006 0.1486190 0.05734944
```

Infection Outcomes and Barriers - Alternative Analysis #1

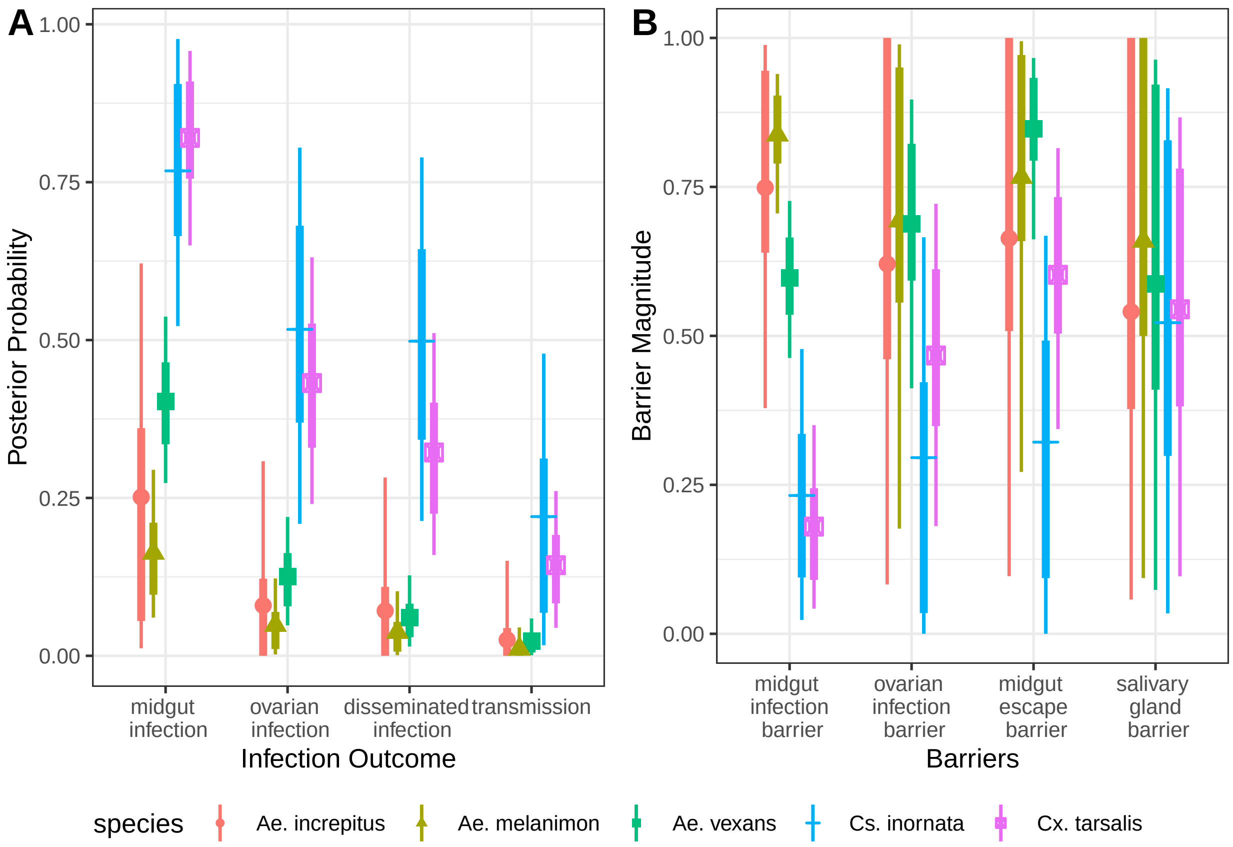

Panel **A** shows the probability of each infection outcome for each species, and panel **B** shows the magnitude of the infection barrier for each tissue, for each species. See **S1 Appendix** for more information about parameter definitions and model fitting procedures.

Alternative Analysis #2

For the following analyses, ovaries that were positive from mosquitoes without detectable virus in legs/wings were considered negative. This applies to 4 of the positive *Ae. vexans* ovaries.

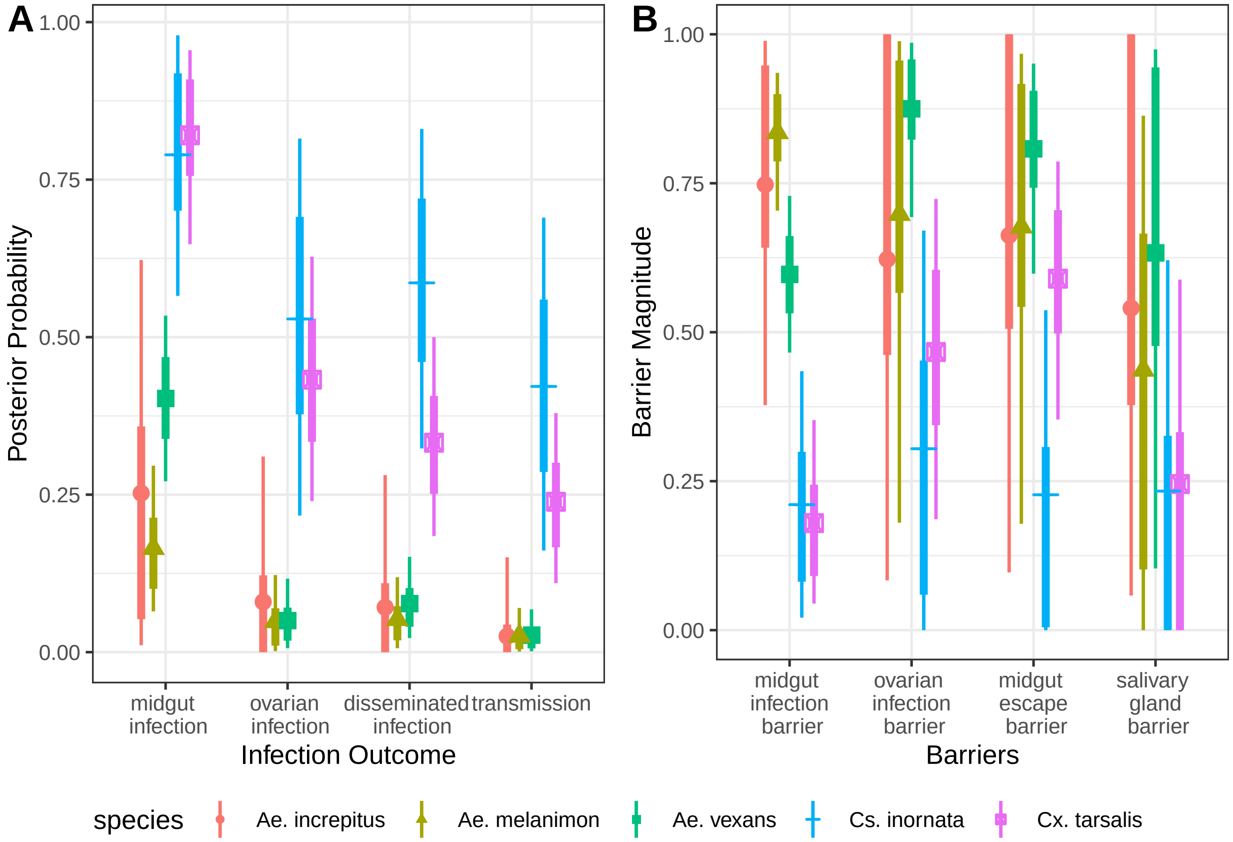

As above, panel **A** shows the probability of each infection outcome for each species, and panel **B** shows the magnitude of the infection barrier for each tissue, for each species. See **S1 Appendix** for more information about parameter definitions and model fitting procedures.

Parameter Summaries for *Ae. vexans*

```
##        Lower95     Median    Upper95       Mean         SD
## a  0.275390146 0.40289803 0.53417685 0.40368829 0.06658457
## b  0.013085263 0.12550200 0.30547355 0.14169392 0.08390816
## c  0.052998937 0.19078416 0.40075977 0.20768868 0.09661910
## d  0.027826541 0.37096523 0.89971571 0.41286773 0.24945202
## p1 0.275390146 0.40289803 0.53417685 0.40368829 0.06658457
## p2 0.005750094 0.05035888 0.11693855 0.05558297 0.03097593
## p3 0.023429198 0.07632249 0.15051906 0.08147943 0.03450876
## p4 0.001384809 0.02720173 0.06754591 0.03046688 0.01922722
```
